# Editorial: Women in Science: Aging and Public Health 2021

**DOI:** 10.3389/fpubh.2022.895113

**Published:** 2022-05-30

**Authors:** Colette Browning, Marcia G. Ory, Xiaomei Pei

**Affiliations:** ^1^Health Innovation and Transformation Centre, Federation University Australia, Ballarat, VIC, Australia; ^2^School of Public Health, Texas A&M University, College Station, TX, United States; ^3^Department of Sociology, Tsinghua University, Beijing, China

**Keywords:** gender, aging adults, public health, women in science, women

Globally there is a recognition that women are under-represented both as scientists and as participants in health research (1–3). In this inaugural “*Women in Science: Aging and Public Health 2021*” Research Topic, we highlight papers from experienced and early career women, and topical areas especially pertinent to older women. While women typically experience greater life expectancy than men, these extra years are often accompanied by more functional limitations and disabilities, although gender-based relationships across the life-course are complex and should not be oversimplified (4–6).

The papers included cover broad public health areas that are timely and relevant to practice and policy. Included are studies from a range of countries including Brazil, Canada, China, France, Germany, Italy, Portugal, Spain, and Sweden. The study methods use national data bases and innovative data linkage, and qualitative interviews. We will briefly highlight the selected papers.

The use of assistive technologies in supporting independence in old age has been promoted as an effective intervention to improve and reduce the costs of care for older people. The adoption of these technologies relies on understanding the needs of older people and responding with acceptable, enabling solutions. Wilkowska et al. in a survey across five countries found that older healthy adults held more positive views toward the use of assistive technologies, while women were more likely than men to see the disadvantages of assistive technologies. Women were also more likely than men to want to age in their own homes and maintain autonomy while receiving care.

A key issue for women as they age is the early identification of health risks and deployment of effective interventions to reduce age-related disability and need for health care. Santos et al. validated the Portuguese version of a health risk screening instrument that measures the 1-year risk of institutionalization, hospitalization, and death in older people. Age predicted risk of all three outcomes and those living alone were more likely to be institutionalized. Interestingly, there were no gender differences in care outcomes.

Other papers used data from large-scale national studies to examine other key issues for older women. Two papers reported studies using the China Health and Retirement Longitudinal Survey database (CHARLS). There is mounting evidence that hearing loss is associated with cognitive impairment in late life. Yuan et al. reported a significant relationship between hearing loss and cognitive impairment and this relationship was attenuated by androgynous gender but not sex. These findings help illuminate psychological mechanisms that contribute to gender-related differences in cognitive health among hearing impaired older adults. Yang and Peng found pain highly prevalent and that women, those living in rural areas, and having physical disabilities were more likely to report pain than their counterparts. The findings can inform health care providers and policy makers.

Little is known about the association between different types of physical activity and cognitive decline in community-residing older adults. Dupré et al. found that those who engaged in more domestic household activities had significantly less cognitive impairment. This 2-year longitudinal study (FRagilité: étude Longitudinale de ses Expressions) advances previous correlational research. It further underscores the importance of examining different types of physical activity for their impact on older adult's functioning, as no relationship was found with leisure or professional activities.

Subjective health ratings are important predictors of morbidity and mortality. Cachioni et al. compared the self-rated health of Brazilian (Frailty in Brazilian Older Adults study) and Portuguese (From Disability to Activity: The Challenge of Aging study) older adults and found that Brazilian older adults were more likely to rate their health as good/very good if they scored low on cognitive impairment and low on neuroticism. Low or intermediate scores on neuroticism were associated with good/very good self rated health. Gender did not impact on these relationships. These findings point to the importance of cross-national research for understanding how health care systems can influence health perceptions and overall health outcomes.

Older adults often have diminished social supports. Linking data from the Canadian Community Health Survey and the hospital discharge records of adults aged 45 years and over living with diabetes, Gupta and Sheng found that those with a weak sense of community attachment were more likely to be hospitalized confirming the role of social factors in health-related outcomes for older people. Gender did not impact on this relationship. Deficits in social engagements amplified by the COVID-19 pandemic have placed older adults with chronic diseases at greater risk for severe complications.

Aging in place where care for older people is provided in their own home is often preferred by the older person, but the burden of care often falls on partners who may be aging themselves and family members. These pressures may influence entry into institutional care. Chammem et al. focus on an interesting approach in Martinique, France, where older people live with foster families who provide care. Qualitative interviews were conducted with older women and men living with foster families and those living in aged care institutions. Foster family care provided more independence, privacy, acceptance of visitors, personalized care and less rules than institutional care, however gender differences in experiences were not explored.

These articles reflect research undertaken by women, while the issues examined are very relevant to aging well. As editors with a special interest in women's health issues, we were surprised that gender was not explicitly studied in many of the papers or that were often no gender differences in study outcomes. In reflection, this reveals a positive broadening of Research Topics by women scientists. Further, the lack of gender differences may reflect methodological issues, but it is also possible that expected relationships are not found cross nationally among older populations. These observations call for further research, especially on the impact of gender roles, and particularly on the impact of the gendered division of labor in the workforce and family, on a variety of health-related outcomes in different settings and populations.

## Author Contributions

CB and MO drafted and finalized the editorial. XP contributed to the drafting of the editorial. All authors contributed to the article and approved the submitted version.

## Conflict of Interest

The authors declare that the research was conducted in the absence of any commercial or financial relationships that could be construed as a potential conflict of interest.

## Publisher's Note

All claims expressed in this article are solely those of the authors and do not necessarily represent those of their affiliated organizations, or those of the publisher, the editors and the reviewers. Any product that may be evaluated in this article, or claim that may be made by its manufacturer, is not guaranteed or endorsed by the publisher.
